# The response of the subthalamo-pallidal networks of the Basal Ganglia to oscillatory cortical input in Parkinson’s disease

**DOI:** 10.1186/1471-2202-15-S1-P57

**Published:** 2014-07-21

**Authors:** Sungwoo Ahn, S Elizabeth Zauber, Robert M Worth, Leonid L Rubchinsky

**Affiliations:** 1 Department of Mathematical Sciences, Indiana University Purdue University Indianapolis, IN 46032, USA; 2 Department of Neurology, Indiana University School of Medicine, Indianapolis, IN 46202, USA; 3 Department of Neurological Surgery, Indiana University School of Medicine, Indianapolis, IN 46202, USA; 4 Stark Neurosciences Research Institute, Indiana University School of Medicine, Indianapolis, IN 46032, USA

## 

Primary motor symptoms of Parkinson’s disease such as bradykinesia and rigidity are associated with increased oscillatory and synchronized activity in the beta frequency band (10-30Hz) throughout the cortical-basal ganglia-thalamic circuitry (see, for example, [1]). The origin of the pathological beta-band oscillations is not completely understood, however it appears that both cortical circuits (reviewed in [2]) as well as subthalamo-pallidal circuitry [3] in the basal ganglia contribute to the generation and expression of pathological rhythmicity. The objective of this study is to explore the oscillatory interactions between cortical and basal ganglia networks in parkinsonian patients and in computational models of Parkinson’s disease.

Two types of data from were recorded from subjects with Parkinson’s disease during the implantation of deep brain stimulation electrodes. We performed microelectrode recordings of neuronal units and local field potentials from the subthalamic nucleus. We also recorded surface EEG from prefrontal and motor areas recorded simultaneously with the recordings in the subthalamic nucleus. The analysis of these data reveals complex patters of correlations between synchrony in cortical circuits (which can be studied noninvasively) and synchrony in the basal ganglia circuits (which requires invasive procedures for investigation).

These results are used to guide the development of computational models to study how activity in the subthalamo-pallidal circuits can be entrained by the oscillatory cortical input. We used conductance-based models for the excitatory-inhibitory networks of neurons from the subthalamic nucleus and the external segment of Globus Pallidus [4,5] and subject them to a series of artificially-generated cortical inputs as well as to the input signals derived from the experimentally recorded EEG.

The biologically-realistic temporal patterns of synchrony, which are generated by the subthalamo-pallidal model network [4] are observed in the networks under cortical inputs too (although for different parameter values). The patterns of responses of beta-band bursting in the model suggest that the experimentally observed beta-band synchronization in Parkinson’s disease may be promoted by the simultaneous action of both cortical and subthalamo-pallidal network mechanisms. This may explain why some theories explaining the origins of pathological beta-band activity in Parkinson’s disease suggest a cortical origin of pathological oscillations, while others suggest local, subthalamo-pallidal generation [1-3].
